# COVID‐19, chronicle of an expected pandemic

**DOI:** 10.15252/emmm.202012463

**Published:** 2020-05-04

**Authors:** Philippe J Sansonetti

**Affiliations:** ^1^ Chaire de Microbiologie et Maladies Infectieuses Collège de France Paris France; ^2^ Institut Pasteur Paris Cedex 15 France

**Keywords:** Microbiology, Virology & Host Pathogen Interaction

## Abstract

What is COVID‐19? What are the causes, parameters, and effects of this disease? What are the short‐ and long‐term prospects? Philippe Sansonetti, Infectious disease specialist and Chief Editor of *EMBO Molecular Medicine*, explains why the fate of the epidemic is in our hands.

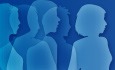

What is COVID‐19? What are the causes, parameters, and effects of this disease? What are the short‐ and long‐term prospects? Philippe Sansonetti, Infectious Disease Specialist and Chief Editor of *EMBO Molecular Medicine*, explains why the fate of the epidemic is in our hands.

*It is difficult to believe in plagues when they fall on your head* (Albert Camus, The plague (1947, Ed. Gallimard)).

The COVID‐19 pandemic is a plague as described by Camus; it is urgent and vital for our society to realize this. It is not too late, but time is running out. It is the third coronavirus‐associated epidemic globally in < 20 years after SARS in 2003 and MERS in 2012. During these two epidemics, we were first worried, then reassured after it was over, and we did not much afterwards in terms of preparedness, therapy, and vaccine. Today, in the absence of treatment and vaccine, the evolution of the COVID‐19 epidemic is nonetheless still in our hands.

Charles Nicolle (1866–1936), professor at the Collège de France and director of the Institut Pasteur in Tunis, wrote in *Destin des maladies infectieuses* (1933):

*So, there will be new diseases. It's a fatal fact. Another fact, also fatal, is that we will never be able to detect them from their origin. Knowledge of infectious diseases teaches men that they are brothers and united. We are brothers because the same danger threatens us, united because contagion comes to us most often from our fellow men. We also, from this point of view and whatever our feelings are towards them, stand together with animals, especially domestic animals*.

He was already predicting the emerging pathogens of the late 20^th^ century and 21^st^ century.

## What are coronaviruses?

Coronaviruses are a huge family of single‐stranded positive RNA viruses. Alpha‐coronaviruses are endemic and infect mammals including humans and cause, for example, mild respiratory and intestinal diseases in children. Beta‐coronaviruses like SARS‐CoV‐2 (the official name of COVID‐19 virus) on the other hand are well adapted to their reservoir, the bat, but not to humans, which explains why human infections are so damaging. Other members of this family, the gamma‐ and delta‐coronaviruses infect birds and fish and, at least for the time being, have not been associated with human disease.

## The identification of the virus

One thing that this epidemic has taught us is that we, scientists now, possess the technology and capabilities to quickly react: As soon as the virus emerged in the City of Wuhan, China, deep sequencing and bioinformatics allowed for the diagnostic of this novel virus in a few days. The viral genomic sequence was then quickly shared and used to develop a detection test based on real‐time PCR for rapid diagnosis and for initiating epidemiological studies all over the planet. Now compare this with the years it took to identify HIV thirty years ago through conventional growing of the virus in *in vitro* culture. Molecular diagnosis has revolutionized this field, and despite the initial delays in communicating about this epidemic, Chinese doctors and biologists quickly reported the first evidence for SARS‐CoV‐2, and provided the first sequences, clearing the way for the global scientific community to further develop diagnostic tools and engage in a race to discover dedicated drugs and vaccines.

## Crossing the species barrier and human responsibility

The name coronavirus comes from the Protein S (S for spike) and its crown‐like shape. The S protein plays a central role for host specificity and pathogenicity as it binds to the ACE2 receptor on the target cell surface and mediates entry of the viral RNA. The phylogenetic tree shows that SARS‐CoV‐2 is very close to SARS 2003 and MERS 2012, and the diseases they cause are very similar. The same is true for their origin: Bats are the main reservoir of coronaviruses. The phylogeny is therefore relatively well known, and one can apply the lessons learned during previous outbreaks, even if no vaccine or therapy is available so far.

SARS‐CoV‐2 is a textbook case of an emergent pathogen after a species jump (“zoonosis”). We have observed these zoonoses for decades, particularly in tropical regions (Ebola is one such example; Marí Saéz *et al*, 2015). Generally, an emergent pathogen can give rise to two scenarios.

1 The virus is ill‐equipped and has little capacity to mutate and therefore to adapt and stabilize in the new species. Human infection is abortive as the virus is not able to establish human‐to‐human transmission. Still, under this scenario, the disease can be very severe as was the avian influenza H5N1 caused by direct bird‐to‐human transmission, reaching ˜60% mortality, but for which limited, if any, human‐to‐human transmission was reported (Wang *et al*, 2008).
2 The virus is better adapted to its new host, thus better able to cross the species barrier, and its adaptation is facilitated by further mutations due to poor fidelity of the enzyme that replicates the positive strand of viral RNA. This is what happened with SARS‐CoV‐2, which jumped relatively easily from bats to humans via an intermediate mammal reservoir. The disease caused by SARS and MERS was more serious, and the mortality rates were higher (10% and 35%, respectively). SARS‐CoV‐2 however is often associated with milder pathologies. This is a typical trade‐off: less virulent, but more transmissible versus more virulent but less transmissible. This balance is extremely important and defines the disease profile.

The natural reservoir is a certain bat species; it is impressive to see how far these animals are capable of carrying these emerging viruses, such as corona. This was the case with Ebola in Africa, and with the Nipah virus in Malaysia, which appeared in the late 1990s. As human behavior changes ecological conditions, these bats increasingly come into contact with animals which are susceptible for a zoonotic species jump and which further replicate the virus. This generates a risk zone around humans, since any contact with these intermediate animals can cause another species jump into humans. In the case of SARS 2003, it is assumed that the intermediate animal was the webbed civet, a feline particularly frequent in Asia; for MERS, it was the camel. For Ebola, it probably was great apes (Malvy *et al*, 2019) while direct bat‐to‐human transmission was also considered (Marí Saéz *et al*, 2015).

For SARS‐CoV‐2, some claim that the intermediate reservoir is the pangolin. Indeed, numerous studies have shown that the virus circulating in pangolins is very close to the one observed in humans, suggesting that the current pandemic results from human behavior and animal trafficking.

We are constantly threatened by these emerging diseases so‐called anthropocene diseases, which are essentially or even exclusively linked to the omnipresent imprint that humans leave on the planet. What is true for the climate and the environment is also true for infectious diseases, in particular emerging ones, and the three are linked.

## Pandemic spread of the virus

This is a story in three acts: (i) stochastic species jumps, (ii) possible transmission to and between human, and (iii) pandemic explosion. It is interesting to note that the distribution map of COVID‐19 clusters and hotspots overlaps with the map of intercontinental air flights (which carried 4 billion passengers in 2019). We can clearly see the role transport plays in the transmission and global spread of these diseases, turning them into pandemics more readily than even before in human history. However, as we speak, more and more cases are being reported in the countries of the intertropical zones, reaching Southern America and Africa where containment will be difficult to achieve.

There are also important environmental aspects linked to the Anthropocene era, such as intensive agriculture and husbandry that profoundly modify ecosystems and cause unanticipated encounters between animal and human habitats, rising temperatures in equatorial regions that drive animal population migration to cooler regions and political instability that causes mass displacement of refugees. Also, different national healthcare systems and a lack of global information exchange and coordination can lead to a delay in diagnosis in some countries, but the comparison of thefts and foci of infection is striking. In how far these effects had an influence on the COVID‐19 pandemic requires further study once the pandemic has passed its apex.

## Parameters of the epidemic

*R0 (basic reproduction rate)* is the average number of secondary infections produced when an infected individual is introduced into a population where all individuals are susceptible. If the R0 is less than 1, there is no epidemic situation; as soon as it is greater than 1, there is an epidemic. In the case of COVID‐19, this number is between 2 and 3. This is therefore a typical epidemic situation. For the Spanish flu of 1918–1919, the R0 was 2.3; tuberculosis is 10 and therefore extremely contagious; and for measles, R0 ranges between 12 and 18.

*The incubation period* is 5–6 days, and all evidences converge to indicate that the patients are contagious from the onset, when they are yet asymptomatic, unlike SARS in 2003 where the contagion only appeared with the peak of viremia after several days after infection. The SARS‐CoV‐2 virus is highly contagious: People transmit while they are still asymptomatic, or just begin to experience mild symptoms.

*The attack rate, the number of newly infected patients compared to the general naive population,* is high, much higher than the seasonal flu. Severe cases are around 10–15% and require hospitalizations that last on average between 7 and 15 days, which is what threatens our health systems.

COVID‐19 is therefore a disease with high epidemic potential that puts a major strain on the healthcare system. This is why governments decided to put in place different strategies to attenuate the progression of the disease.

*The mortality rate* is relatively low. When we will be able to get a full picture of this pandemic, we will most likely notice that it was between 1 and 2%. It seems higher during periods of exponential spread as is currently the case in the USA, France, Spain, or Italy. Not necessarily because the disease is more severe during this period, but likely because death count is indisputable, while it is difficult to assess the number of infections, which is always higher than observed. Undoubtedly, the majority of infected people develop a mild form of this disease, which makes accurate calculation of the mortality rate difficult at present. At the time of publication, mass serological testing is being rolled out in countries such as Germany, particularly in hotspot areas, which will give a much more accurate estimate of mortality rates. But the mortality rate increases due to the stress on the healthcare system and the availability of hospital beds. 1% mortality, 10% of severe cases, those are not large numbers statistically speaking, but compared to the number of infections, and taking into account the transmissibility and infectivity of the virus, we are reaching a situation that endangers our healthcare system. This is what legitimizes the flatten‐the‐curve policy. Yet, one good news: children younger than 10 years‐old are mostly not ill even though they can get infected and transmit the virus.

## Eco‐pathology of beta‐coronaviruses

The receptor for SARS‐CoV‐1 and SARS‐CoV‐2, angiotensin II converting enzyme (ACE2), is an enzyme attached to the surface of cells, including lungs, pneumocytes and endothelium, endocardium, kidney, liver, and intestine. It came as a surprise that the virus binds to an important enzyme for the regulation of blood pressure. This could explain the severity of the disease in the respiratory tract with instances of pneumonia and ultimately, acute respiratory distress syndrome (ARDS) observed in elderly subjects or individuals with chronic co‐morbidities (i.e., diabetes, hypertension, cardio‐respiratory insufficiency, chronic immunosuppression). The virus targets the blood–lung barrier, affecting the oxygen exchange zone.

Acute respiratory distress syndrome has classic inflammatory signs, a so‐called “cytokine storm” characterized by a significant increase in cytokines and proinflammatory chemokines. What is less typical for corona infections is the destruction of the alveolo‐capillary barrier, which seldom happens also in COVID‐19, but when it does, requires fast intervention. ARDS can also occur in younger individuals during the healing phase, which, in this case though, may be linked to the immune response.

The adaptive immune response specific to the virus is still poorly understood. We know that this virus affects IFN‐driven immunity, but we do not know why, or which effectors blunt this immune response. It is possible that the virus has acquired this strategy in bats that express constitutively low level of IFN. Further work is urgently needed to learn to control the disease efficiently and sustainably.

## The future is in our hands

The future of the COVID‐19 pandemic is in our hands. So far, we can only rely on prevention and symptomatic treatment of severe forms. Regarding prevention, health authorities have three main options. The first—which may seem cynical—considers that the more people become infected, the quicker the population will become immune until the epidemic peters out as the virus finds no more immunologically naive individuals. This is the principle of group/herd immunity. If 60% of the population were infected by SARS‐CoV‐2, the epidemic will stop. But this would come at the cost of a very brutal epidemic peak with a high number of severe cases and deaths. We saw it during the epidemic of the Asian flu in the United Kingdom in 1957. For a week to ten days, the health system imploded, because the health personnel were sick, the equipment insufficient, and the number of seriously ill patients exploded. Totally opposite is the “Chinese approach”: to isolate cities and individuals, which undeniably appears effective in controlling the epidemic. The risk is that because only few individuals have been infected, the attack rate was reduced due to strict confinement, hence many individuals are still immunologically naive and vulnerable to a second viral wave and risk an epidemic rebound. The intermediate option, which has been taken in most of European countries, is to flatten the curve over time with different levels of isolation and social distancing, hoping that even if less than 60% of the population will be infected, above all, the healthcare system will hopefully be preserved. The approach is based on a concept of social distancing (two meters/6 feet apart) and individual hygiene. It seems that in addition to airborne contamination, contaminated hands are also a major vector, either by direct contact with an infected patient, or indirectly by contact with a contaminated surface on which the virus seems to be able to survive for many hours. So, no kiss, no hug, no handshake and absolute hand hygiene. Avoid touching face unless hands have been thoroughly washed with soap or decontaminated with a hydro‐alcoholic gel. The importance of wearing masks for the general population is still debated in some countries that quickly ran short of these masks. In agreement with attitudes in Asian countries, wearing masks is now strongly advised to complement hygiene measures. Common sense starts to prevail.

Unfortunately, the measures that have been implemented so far have been clearly insufficient, as we have seen in Italy, Spain, France, UK and as we now see in the USA. Confinement and isolation at home have therefore been decided for an extended period at the cost of a huge economic risk, given the intrinsic dynamics of the epidemic. Failure of these measures in these countries will have to be compared with their success in Asian countries like South‐Korea, Taiwan, Singapore, and to a certain extent in Germany. Large implementation of diagnostic combined with isolation of SARS‐CoV‐2 positive individuals may be part of the answer. No matter the strategy taken by health authorities, we need to convince ourselves that our fate is in our hands. We stepped into another world in only a few weeks. What we valued yesterday—our daily activities, our hobbies, our work—must be weighted in relation to the gravity of the situation.

## Treatments

Finally, it is essential to find antiviral treatments for severely affected patients and to block transmissibility from individual to individual. A few options are as follows: (i) “repositioning” of drugs already tested for other viruses, such as HIV, or drugs that are already commercially available; (ii) better understanding of the pathophysiology of ARDS in order to develop a dedicated pharmacology using repositioned and novel molecules; and (iii) developing an effective vaccine. The risk of rebounds justifies the absolute necessity of a vaccine that is the only way to achieve durable elimination of COVID‐19 and even eradication of SARS‐CoV‐2, including in low‐income countries with fragile health systems. Design, development, validation, clinical studies, and registrations with regulatory agencies takes between 8 and 12 years on average for a standard vaccine. This is incompatible with the urgency of an emerging epidemic like COVID‐19. Fortunately, novel paradigms of vaccine discovery and development are on their way to considerably shorten the delays. Nonetheless, we know that we would not have a vaccine available until at least next year. In the meantime, in spite of enormous difficulties, we need a global mobilization for more basic and applied research from academia and the pharmaceutical and vaccine industry. As much as we have been able to massively reduce the time to identify novel pathogens, we need to reduce the time to develop the tools of their control. Again, our future is in our hands.

Adapted from an article published in French in laviedesidees.fr, March 2020.

## References

[emmm202012463-bib-0001] Malvy D , McElroy AK , de Clerck H , Günther S , van Griensven J (2019) Ebola virus disease. Lancet 393: 936–948 30777297 10.1016/S0140-6736(18)33132-5

[emmm202012463-bib-0002] Marí Saéz A , Weiss S , Nowak K , Lapeyre V , Zimmermann F , Düx A , Kühl HS , Kaba M , Regnaut S , Merkel K *et al* (2015) Investigating the zoonotic origin of the West African Ebola epidemic. EMBO Mol Med 7: 17–23 25550396 10.15252/emmm.201404792 PMC4309665

[emmm202012463-bib-0003] Wang H , Feng Z , Shu Y , Yu H , Zhou L , Zu R , Huai Y , Dong J , Bao C , Wen L *et al* (2008) Probable limited person‐to‐person transmission of highly pathogenic avian influenza A (H5N1) virus in China. Lancet 371: 1427–1434 18400288 10.1016/S0140-6736(08)60493-6

